# Comparison of Diagnostic Yield and Safety between Semirigid Pleuroscopic Cryobiopsy and Forceps Biopsy for Undiagnosed Pleural Effusion

**DOI:** 10.1155/2019/5490896

**Published:** 2019-12-17

**Authors:** Chung-Shu Lee, Shih-Hong Li, Chih-Hao Chang, Fu-Tsai Chung, Li-Chung Chiu, Chun-Liang Chou, Chih-Wei Wang, Shu-Min Lin

**Affiliations:** ^1^Department of Thoracic Medicine, Chang Gung Memorial Hospital, Chang Gung University, School of Medicine, Taipei, Taiwan; ^2^Division of Pulmonary and Critical Care, Department of Internal Medicine, Saint Paul's Hospital, Taoyuan City, Taiwan; ^3^Department of Thoracic Medicine, Taipei Medical University Hospital, Taipei Medical University, School of Medicine, Taipei, Taiwan; ^4^Department of Anatomic Pathology, Chang Gung Memorial Hospital, Linkou Branch, Taoyuan City, Taiwan; ^5^Department of Respiratory Therapy, Chang Gung Memorial Hospital, Chang Gung University, College of Medicine, Taoyuan, Taiwan

## Abstract

For undiagnosed pleural effusion, diagnostic yields and safety were similar between pleuroscopic cryobiopsy and forceps biopsy, but cryobiopsy obtained a larger pleural tissue sample than forceps biopsy.

## 1. Background

Pleural effusion is a common problem in medical practice. Undiagnosed pleural effusion is frequently encountered, even after thoracentesis and closed pleural biopsy [1, 2]. Patients with pleural effusion should undergo thoracoscopy to establish a diagnosis. Surgical thoracoscopy, widely known as video-assisted thoracoscopic surgery (VATS), is frequently employed for making such a diagnosis. Recent evidence suggests that another diagnostic tool with a semirigid pleuroscope is an effective diagnostic tool for undiagnosed pleural effusion. Semirigid pleuroscopy is conducted by nonsurgeon pulmonologists in the endoscopy suite, with patients under local anesthesia and conscious sedation. The diagnostic sensitivity of rigid pleuroscopy ranges from 85% to 93% [3]. By contrast, semirigid pleuroscopy has 91% sensitivity and 100% specificity, with comparable efficacy to surgical thoracoscopy [4].

Semirigid pleuroscopy enables the examination of the pleural lesion and guides the biopsy of abnormal pleural lesions. Pleuroscopic biopsy with flexible forceps is limited by the size of the forceps used. Due to inadequate mechanical strength, forceps biopsy is associated with concerns regarding obtaining pleural tissue of adequate size and depth [5]. In the bronchoscopic procedure, cryobiopsy is an efficient method for obtaining larger specimens and may avoid crushed artifact, which is a feature of forceps biopsy [6, 7]. A recent study revealed that the diagnostic yield of cryobiopsy under semirigid pleuroscopy is comparable to that of conventional pleural biopsy using flexible forceps, and it provides larger and deeper tissue samples with a more favorably preserved cellular architecture [8]. In addition, pleuroscopic cryobiopsy is a safe procedure without major adverse events or substantial bleeding [8].

The aim of this study was to compare the diagnostic yield, safety, and clinical outcomes between cryobiopsy and forceps biopsy performed under semirigid pleuroscopy.

## 2. Methods

**Figure 1:**
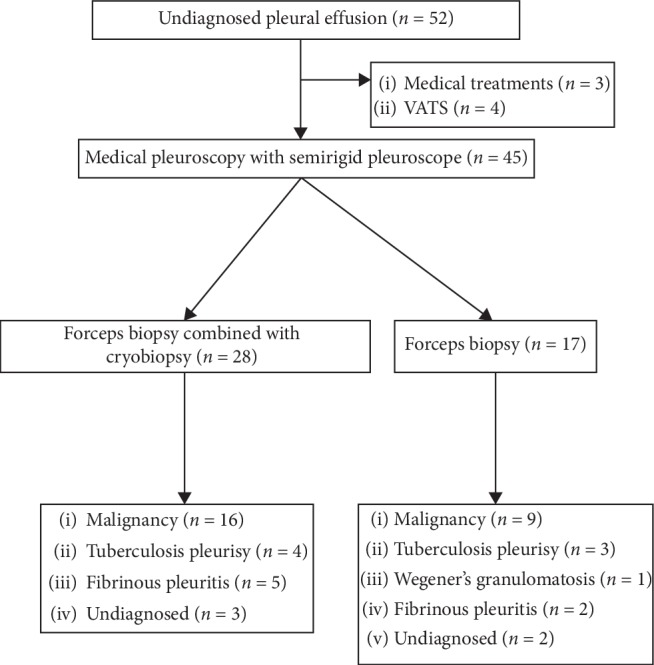
Diagnosis of patients who received pleuroscopic cryobiopsy and flexible forceps biopsy.

This study was approved by the Institutional Review Board of Chang Gung Memorial Foundation (IRB No. 201700671B0). This study retrospectively recruited 45 patients from April 2016 to June 2017 from Linkuo Chang Gung Memorial Hospital, Taiwan, a tertiary referral medical center (Figure 1). Consecutive patients who received semirigid pleuroscopy with forceps biopsy and cryoprobe pleural biopsy within the study period were identified from the database, and their demographics characteristics were recorded.

### 2.1. Procedure and Equipment

In all patients, pleuroscopic examination was conducted by an experienced operator and two trained assistants. Additionally, during the procedure, all patients received moderate sedation with fentanyl and midazolam with or without propofol. Before the initiation of the procedure, thoracic ultrasound was performed to determine the optimal entry point. After local administration of 2% lidocaine for anesthesia, a 1.0 cm skin incision was made followed by thoracotomy with an 8 mm flexible trocar (MAJ-1058; Olympus Medical Systems Corp., Japan). A semirigid thoracoscope (LTF-240; Olympus, Tokyo, Japan) was used to drain the effusion initially and explore the pleural cavity. Accessible pleural space would be entirely visualized first, and the next step was to determine the biopsy site. Forceps (FB-15C-1; Olympus) or a cryoprobe was used for biopsy through the working channel of a thoracoscope. A 1.9 mm flexible cryoprobe (Erbokryo CA; Erbe, Germany) was used to perform cryobiopsy with cryogen (carbon dioxide). The cooling time for each cryobiopsy was approximately 3 seconds [9], and the temperature of the probe tip reached approximately −70°C. The pleuroscope and cryoprobe with biopsied pleural tissue attached were withdrawn simultaneously. The cryoprobe and biopsy samples were placed in normal saline at room temperature to thaw, enabling the tissue to detach from the cryoprobe. Each cryobiopsy included one freeze-thaw cycle except for the hard pleura. At least three biopsies was our standard procedure in each patient routinely. The biopsy area was restricted in parietal pleura. For histopathological analyses, all biopsy samples were transported in separate formalin containers. The biopsy samples were processed according to standard protocols for histopathology and immunohistochemical staining. After the biopsies were completed, a drainage tube with a 16 Fr pigtail catheter (BT-PDS-1630-W-NK1; Bioteq, Taipei, Taiwan) was placed into the pleural cavity to monitor and drain the pleural effusion. The patients received a follow-up chest X-ray in 3 hours after the procedure to make sure the location of the drainage tube. The next follow-up chest X-ray was arranged 3 days later. If follow-up chest radiography did not reveal pneumothorax and the daily drainage amount was less than 50 ml, the catheter was removed.

### 2.2. Clinical Outcomes

All patients who did not have a specific etiological diagnosis were followed up for a minimum of 6 months. Fibrinous pleuritis was accepted as the final diagnosis in patients with compatible histopathology and no evidence of malignancy or an alternative diagnosis at the end of follow-up [8]. A pathologist who was blinded to the study design measured the tissue sample size. Postbiopsy bleeding was defined as mild if it was self-limiting, moderate if electrocautery application was required for hemostasis, and severe if intravenous resuscitation, blood transfusion, and surgical or radiological interventions were required for its management [8].

### 2.3. Statistical Analysis

Student's *t*-test was used for comparisons of normally distributed variables between the cryobiopsy group and the forceps biopsy group. Categorical variables were analyzed using the chi-squared test. Categorical variables are expressed as frequencies and percentages, and continuous variables are presented as the mean ± standard deviation. All statistical analyses were performed using SPSS software version 18.0 (SPSS Inc., Chicago, IL, USA). Two-tailed *p* values less than 0.05 were considered statistically significant.

## 3. Results

This study included a total of 45 patients admitted to the Linkuo Branch of Chang Gung Memorial Hospital in Taiwan from April 2016 to June 2017. Under semirigid pleuroscopy, 28 patients received cryobiopsy and 17 patients received forceps biopsy. Their demographic data are provided in Table 1. The average age of all patients was 64.4 years, and two-thirds of patients were men. No differences were observed in both groups regarding the smoking status, location of pleural effusion, and biochemical data except for the LDH level in pleural effusion.

### 3.1. Diagnostic Yield and Diagnosis

Patients were diagnosed with malignancy, tuberculosis pleurisy, Wegener's granulomatosis, or fibrinous pleuritis. The diagnostic rate was 89.3% and 88.2% in the cryobiopsy and forceps biopsy groups, respectively (Table 2). The length of the tissue sample was 9.1 ± 5.7 mm and 5.3 ± 3.8 mm in the cryobiopsy and forceps biopsy groups, respectively (*p*=0.020). The area of the tissue sample was larger in the cryobiopsy group than in the forceps biopsy group (56.0 ± 61.3 mm^2^ vs. 10.2 ± 14.3 mm^2^, respectively; *p*=0.001). The volume of the tissue sample was larger in the cryobiopsy group than in the forceps biopsy group (232.1 ± 411.4 mm^3^ vs. 13.4 ± 22.6 mm^3^, respectively; *p*=0.009). In patients who failed to reach diagnosis after pleuroscopy, there were no statistical differences in length, area, and volume of tissue samples compared with those with definite diagnosis after pleuroscopy (Table 3).

### 3.2. Safety Results and Clinical Outcomes

Neither moderate nor severe bleeding was observed during the procedure or the following clinical course. Three patients in the cryobiopsy group and one patient in the forceps biopsy group developed mild bleeding. There was no subcutaneous emphysema, pneumomediastinum, pneumothorax, or wound infection developed in both groups. Among all patients who received pleuroscopy, no vessels, nerve, muscles, or bones were seen in the histopathological finding of the pleural samples. The median hospital stay after pleuroscopy was similar in the cryobiopsy group (11 days; IQR, 8–14.5) and forceps biopsy group (11 days; IQR, 7–18; *p*=0.916) (Table 2). In this study, all the patients received chest computed tomography and chest echogram before the pleuroscopic procedure. Therefore, no trapped lung was noted in the study population.

## 4. Discussion

This study demonstrated that pleuroscopic cryobiopsy had a similar diagnostic yield to flexible forceps biopsy. There was no major complication developed in both groups. Larger samples were obtained through cryobiopsy than through forceps biopsy. In patients who failed to reach diagnosis after pleuroscopy, there were no statistical differences in length, area, and volume of tissue samples compared with those with definite diagnosis after pleuroscopy.

In our study, pleuroscopy with either cryobiopsy or forceps biopsy provided diagnostic yields of more than 88% in undiagnosed pleural effusion; the rate is consistent with that reported in previous studies [3]. Recent studies have revealed that cryobiopsy pleural samples were significantly larger, deeper, and with fewer crushed artifacts than those obtained using forceps biopsy [8]. Similarly, this study also demonstrated that larger pleura samples were obtained through cryobiopsy than through forceps biopsy. Although studies have identified no relationship between sample size and diagnostic yield [10], obtaining larger tissue may be crucial in establishing histological diagnosis or performing the mutation assay in patients with malignant pleural effusions. Recently, rebiopsies are frequently performed in relapse of advanced non-small-cell lung cancer. The results of rebiopsies may be used to predict therapeutic resistance and redirect the selection of subsequent target therapies [11]. Considering the high diagnostic yield and safety profile, pleuroscopic biopsy may be a useful tool for rebiopsy in non-small-cell lung cancer patients with pleural effusion. In addition, our results revealed that, in patients who received pleuroscopy, no vessels, nerve, muscles, or bones were seen in the histopathologic finding of the pleural samples. The high purity of pleural tissue obtained by this procedure may provide good reliability of the mutation assay.

Common complications of bronchoscopic cryobiopsy are bleeding, pneumothorax, and pneumomediastinum. In this study, none of the patients developed pneumothorax or pneumomediastinum after procedures were completed. Regarding complications of pleuroscopy-associated bleeding, none of the patients developed moderate or major bleeding. Only 9% of patients experienced mild bleeding in our study group. Therefore, in addition to its remarkable diagnostic yield, pleuroscopic biopsy with cryobiopsy and forceps biopsy is a relatively safe procedure performed by experienced operators.

Semirigid pleuroscopy is a minimally invasive procedure for the examination and biopsy of the pleural space. The procedure is performed by pulmonologists in a spontaneously breathing patient under moderate sedation in a bronchoscopy suite [4]. Other options for examining undiagnosed pleural effusion are surgical thoracoscopic procedures such as thoracoscopy and VATS with the multiple- or uniport technique. Under thoracoscopy, physicians can use the scopes and a single-port incision to inspect, biopsy, and perform thoracic procedures. By introducing video-assisted cameras offering a panoramic view of the hemithorax instead of the previous tunnel-like view with direct vision, VATS is commonly used in surgical practice [12]. Initially, the VATS procedures employ a multiport approach, with one port used for visualization and additional ports for instrumentation. Due to these advances, VATS has become the standard care for many procedures. As this technique was further developed, the traditional multiport approach has evolved into a uniportal approach [13]. In uniportal VATS, the placement of the surgical instruments and that of the camera are both done through the same incision.

First thoracoscopy was performed by Jacobaeus in 1910 [14]. VATS further developed with the availability of endoscopic linear mechanical staplers and thoracoscopic cameras from thoracoscopy [15]. Conventional VATS included 3-4 ports, and some studies have reported using a uniport approach during surgery [16]. VATS could be performed with local anesthesia and sedation [17]. Compared with surgical thoracoscopy, besides the cost [18], the advantage of semirigid pleuroscopy is that it can be performed in patients with poor lung function or those with a high anesthetic risk associated with surgical procedures. A previous study reported that medical pleuroscopy is safe in patients with a preoperative forced expiratory volume in 1 second <1 L in the pulmonary function test and patients who exhibit a poor performance status [19]. Therefore, semirigid pleuroscopy may be a favorable diagnostic tool for selective patients unsuited to VATS under general anesthesia. In our study, 4 patients with unknown pleural effusion received VATS because of the need of adhesiolysis for pleural adhesion and septum formation detected by chest ultrasound. Due to the single-port nature of a semirigid pleuroscope, limited pleural visualization and range of biopsy are common problems in patients with marked pleural adhesion or septum formation. Therefore, VATS may be a better choice for patients with pleural effusion and marked adhesion and septum formation.

This study demonstrated that sample size could not explain the failure to reach diagnosis by semirigid pleuroscopic biopsy. These patients were clinical monitored for more than 6 months to exclude malignancy or uncontrolled infection. Although the diagnostic yield of pleuroscopic biopsy may have its own limitation, patients without final diagnosis after pleuroscopy need continuous monitoring [20]. In addition, VATS may be another option for those without final diagnosis after receiving pleuroscopy.

This study has several limitations: First, its major limitation is its retrospective design, which may have led to bias in patient selection or statistical analysis. Second, the sample size of the study was small; therefore, the results of the study should be interpreted with caution. To further confirm the results of this study, a prospective study with a larger sample size is required to evaluate the utility of semirigid pleuroscopy in patients with undiagnosed pleural effusion.

## 5. Conclusions

This study demonstrated that semirigid pleuroscopy is a relatively safe procedure with a low complication rate. Larger pleural tissue specimens were obtained through pleuroscopic cryobiopsy than through forceps biopsy, although both types of biopsies provided similar diagnostic yields regarding undiagnosed pleural effusion.

## Figures and Tables

**Table 1:** Baseline characteristics of the study population.

Variables	Total, *n*=45	Cryobiopsy, *n*=28	Forceps biopsy, *n*=17	*p* value
Age, median (IQR)	64.4 (55.4–76.4)	64.6 (55.3–73.4)	64.4 (55.4–77.6)	0.590
Male gender	30 (66.7%)	21 (75.0%)	9 (52.9%)	0.128
Smoking status				
Currently smoke	7 (15.6%)	3 (10.7%)	4 (23.5%)	0.250
Had quit smoke	5 (11.1%)	5 (17.9%)	0 (0.0%)	0.065
Never smoke	32 (71.1%)	19 (67.9%)	13 (76.5%)	0.537
Location of pleural effusion				
Right	20 (40.0%)	10 (35.7%)	10 (58.8%)	0.130
Left	18 (40.0%)	14 (50.0%)	4 (23.5%)	0.079
Bilateral	7 (15.6%)	4 (14.3%)	3 (17.6%)	0.763
Pleural effusion LDH (mg/dL), median (IQR)	394 (187–956)	635 (275.3–1290.3)	273 (143–501.5)	0.030
Pleural effusion total protein (mg/dL), median (IQR)	4.5 (4.0–5.2)	4.6 (4.0–5.1)	4.5 (3.9–5.3)	1.000
Serum LDH (mg/dL), median (IQR)	241.5 (199.8–315.5)	242 (228.3–307.8)	215.5 (179.8–321.2)	0.370
Serum total protein (mg/dL), median (IQR)	6.8 (5.7–7.1)	6.9 (6.3–7.2)	6.2 (5.3–6.8)	0.081
Pleural effusion/serum LDH ratio	2.49 ± 4.38	3.16 ± 5.46	1.42 ± 1.20	0.333
Pleural effusion/serum total protein ratio	0.67 ± 0.13	0.66 ± 0.14	0.67 ± 0.12	0.787

**Table 2:** Final diagnoses and complications.

	Cryobiopsy, *n*=28	Forceps biopsy, *n*=17	*p* value
Sample pieces	7 (3.5–8)	6 (5–7.5)	0.686
Tissue size			
Length (mm)	9.1 ± 5.7	5.3 ± 3.8	0.020
Area (mm^2^)	56.0 ± 61.3	10.2 ± 14.3	0.001
Volume (mm^3^)	232.1 ± 411.4	13.4 ± 22.6	0.009
Bleeding			
Moderate/severe	0	0	—
Mild	3	1	0.581
No bleeding	25	16	0.581
Subcutaneous emphysema	0	0	
Pneumothorax	0	0	
Pneumomediastinum	0	0	
Wound infection	0	0	
Diagnostic rate	89.3% (25/28)	88.2% (15/17)	0.913
Hospital stay after pleuroscopic biopsy (days), median (IQR)	11 (8–14.5)	11 (7–18)	0.916
Final diagnosis			
Malignancy	16 (57.1%)	9 (52.9%)	
Tuberculosis pleurisy	4 (14.3%)	3 (17.6%)	
Fibrinous pleuritis	5 (17.9%)	2 (11.8%)	
Wegener's granulomatosis	0 (0%)	1 (5.9%)	
Undiagnosed	3 (10.7%)	2 (11.8%)	

**Table 3:** Comparison of tissue size between the diagnosed group and the undiagnosed group.

	Diagnosed, *n*=40	Undiagnosed, *n*=5	*p* value
Tissue size			
Length (mm)	7.5 ± 5.50	8.9 ± 4.72	0.590
Area (mm^2^)	38.0 ± 55.7	44.4 ± 36.8	0.805
Volume (mm^3^)	153.5 ± 357.9	116.5 ± 144.0	0.821

## Data Availability

No data were used to support this study.

## References

[1] Poe R. H., Israel R. H., Utell M. J., Hall W. J., Greenblatt D. W., Kallay M. C. (1984). Sensitivity, specificity, and predictive values of closed pleural biopsy. *Archives of Internal Medicine*.

[2] Prakash U. B. S., Reiman H. M. (1985). Comparison of needle biopsy with cytologic analysis for the evaluation of pleural effusion: analysis of 414 cases. *Mayo Clinic Proceedings*.

[3] Rahman N. M., Ali N. J., Brown G. (2010). Local anaesthetic thoracoscopy: British Thoracic Society pleural disease guideline 2010. *Thorax*.

[4] Agarwal R., Aggarwal A. N., Gupta D. (2013). Diagnostic accuracy and safety of semirigid thoracoscopy in exudative pleural effusions. *Chest*.

[5] Ofiara L. M., Navasakulpong A., Ezer N., Gonzalez A. V. (2012). The importance of a satisfactory biopsy for the diagnosis of lung cancer in the era of personalized treatment. *Current Oncology*.

[6] Chang C. H., Lee C. S., Li S. H. (2017). Feasibility of radial endobronchial ultrasound-guided bronchoscopic cryobiopsy without fluoroscopy for lung parenchymal lesions. *Canadian Respiratory Journal*.

[7] Franke K.-J., Theegarten D., Hann von Weyhern C. (2010). Prospective controlled animal study on biopsy sampling with new flexible cryoprobes versus forceps: evaluation of biopsy size, histological quality and bleeding risk. *Respiration*.

[8] Thomas R., Karunarathne S., Jennings B. (2015). Pleuroscopic cryoprobe biopsies of the pleura: a feasibility and safety study. *Respirology*.

[9] Pathak V., Shepherd R. W., Hussein E., Malhotra R. (2017). Safety and feasibility of pleural cryobiopsy compared to forceps biopsy during semi-rigid pleuroscopy. *Lung*.

[10] Dhooria S., Singh N., Aggarwal A. N., Gupta D., Agarwal R. (2014). A randomized trial comparing the diagnostic yield of rigid and semirigid thoracoscopy in undiagnosed pleural effusions. *Respiratory Care*.

[11] Jekunen A. P. (2015). Role of rebiopsy in relapsed non-small cell lung cancer for directing oncology treatments. *Journal of Oncology*.

[12] Migliore M., Deodato G. (2000). Thoracoscopic surgery, video-thoracoscopic surgery, or VATS: a confusion in definition. *The Annals of Thoracic Surgery*.

[13] Migliore M. (2003). Efficacy and safety of single-trocar technique for minimally invasive surgery of the chest in the treatment of noncomplex pleural disease. *The Journal of Thoracic and Cardiovascular Surgery*.

[14] Marchetti G. P., Pinelli V., Tassi G. F. (2011). 100 years of thoracoscopy: historical notes. *Respiration*.

[15] Gonzalez-Rivas D. (2016). Uniportal thoracoscopic surgery: from medical thoracoscopy to non-intubated uniportal video-assisted major pulmonary resections. *Annals of Cardiothoracic Surgery*.

[16] Gonzalez-Rivas D., Paradela M., Fernandez R. (2013). Uniportal video-assisted thoracoscopic lobectomy: two years of experience. *The Annals of Thoracic Surgery*.

[17] Katlic M. R., Facktor M. A. (2010). Video-assisted thoracic surgery utilizing local anesthesia and sedation: 384 consecutive cases. *The Annals of Thoracic Surgery*.

[18] McDonald C. M., Pierre C., de Perrot M. (2018). Efficacy and cost of awake thoracoscopy and video-assisted thoracoscopic surgery in the undiagnosed pleural effusion. *The Annals of Thoracic Surgery*.

[19] Migliore M., Giuliano R., Aziz T., Saad R. A., Sgalambro F. (2002). Four-step local anesthesia and sedation for thoracoscopic diagnosis and management of pleural diseases. *Chest*.

[20] DePew Z. S., Verma A., Wigle D., Mullon J. J., Nichols F. C., Maldonado F. (2014). Nonspecific pleuritis: optimal duration of follow-up. *The Annals of Thoracic Surgery*.

